# Multimodality Imaging in Cardiac Amyloidosis

**DOI:** 10.3390/jcm15010163

**Published:** 2025-12-25

**Authors:** Mayuresh Chaudhari, Mahi Lakshmi Ashwath

**Affiliations:** 1 UT Health Sciences Centre, Glen Biggs Institute, San Antonio, TX 78229, USA; 2 UHS/UT Heart and Vascular Institute, San Antonio, TX 78229, USA

**Keywords:** cardiac amyloidosis, echocardiography, cardiac MRI, nuclear imaging, transthyretin (ATTR) amyloidosis, amyloid light chains (AL) amyloidosis, multimodality imaging

## Abstract

Cardiac amyloidosis is an underdiagnosed cause of heart failure characterized by extracellular deposition of misfolded proteins. Advances in non-invasive imaging, including echocardiography, cardiac magnetic resonance imaging (CMR), and radionuclide imaging, have significantly enhanced the diagnostic accuracy and monitoring of cardiac amyloidosis. This review explores the role of each modality, their individual strengths, and current consensus recommendations. Emphasis is placed on the integration of multimodal imaging to guide diagnosis, prognosis, and therapeutic decisions in both AL and ATTR amyloidosis.

## 1. Introduction

Cardiac amyloidosis is a progressive and frequently underrecognized cause of heart failure, resulting from the deposition of misfolded protein fibrils within the myocardium [1,2]. Once considered a rare diagnosis, advances in non-invasive imaging and improved clinical awareness have led to increased recognition of this condition, particularly in patients with heart failure with preserved ejection fraction (HFpEF) [3,4]. Over the past decade, cardiac amyloidosis has shifted from an uncommon and often overlooked diagnosis to a condition increasingly identified across diverse cardiovascular populations, driven by the growing understanding that transthyretin amyloidosis is far more prevalent—especially among older adults—and that both AL and ATTR forms frequently mimic more common cardiac diseases. The availability of sensitive monoclonal protein assays, emerging targeted therapies, and the recognition of early extramyocardial manifestations have reinforced the urgent need for timely detection and accurate subtype classification. The two primary forms of cardiac amyloidosis are light-chain (AL) amyloidosis, caused by plasma cell dyscrasias, and transthyretin (ATTR) amyloidosis, which occurs in either a hereditary (ATTRv) or wild-type (ATTRwt) form [5,6]. Early and accurate diagnosis is crucial, as treatment options and prognoses vary significantly between subtypes [7]. Historically, diagnosis relied heavily on tissue biopsy and invasive methods; however, the emergence of advanced cardiac imaging modalities—namely echocardiography, cardiac magnetic resonance imaging (CMR), and radionuclide imaging using bone-avid tracers—has revolutionized the diagnostic approach [8,9,10]. These tools now enable non-invasive detection, precise classification, and longitudinal monitoring of cardiac involvement, while also supporting early identification when therapeutic interventions are most effective. This review highlights the pathophysiology of cardiac amyloidosis and evaluates the role of each imaging modality in diagnosis, risk stratification, and disease monitoring. A multimodality approach, integrating findings from echocardiography, CMR, and nuclear imaging, is increasingly regarded as the gold standard for comprehensive assessment [11,12].

## 2. Pathophysiology of Cardiac Amyloidosis

Amyloidosis is a disorder characterized by the extracellular deposition of misfolded protein fibrils, which adopt a beta-pleated sheet conformation and disrupt normal tissue architecture and function [13]. In the heart, these deposits increase myocardial stiffness, impair relaxation, and eventually lead to restrictive cardiomyopathy and heart failure [14].

AL amyloidosis results from a clonal proliferation of plasma cells producing excessive light chains (kappa or lambda), which misfold and deposit in various organs including the heart. Cardiac involvement is a major determinant of prognosis in AL amyloidosis, with median survival less than six months in untreated patients presenting with heart failure symptoms [15].

**Figure 1:**
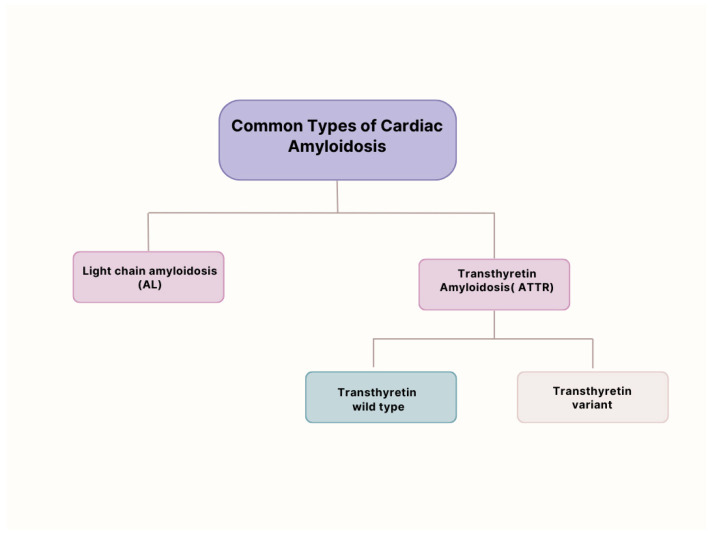
Figure illustrating types of amyloidosis.

ATTR amyloidosis is caused by the misfolding of transthyretin, a tetrameric protein synthesized primarily by the liver. It transports thyroxine and retinol-binding protein. Two subtypes exist (Figure 1):

- Wild-type ATTR (ATTRwt): Formerly known as senile systemic amyloidosis, this form predominantly affects elderly males and is increasingly recognized as a cause of HFpEF [16,17].
- Variant ATTR (ATTRv): An autosomal dominant condition caused by mutations in the *TTR* gene, leading to earlier and more aggressive cardiac involvement [18].

Both forms result in progressive amyloid infiltration of the myocardium, leading to diastolic dysfunction, conduction system abnormalities, and arrhythmias [19]. Histologically, the myocardium in amyloidosis demonstrates widespread interstitial amyloid deposits with myocyte atrophy and minimal inflammation. The pattern and extent of amyloid distribution differ between AL and ATTR, influencing imaging characteristics and clinical presentation [20].

Recent studies underscore the need for prompt diagnosis and differentiation between AL and ATTR, as specific therapies—ranging from chemotherapeutic agents to transthyretin stabilizers and gene silencers—are now available, making early identification imperative [21,22].

## 3. Clinical Presentation and Diagnostic Evaluation

Cardiac amyloidosis commonly presents with nonspecific symptoms, making clinical diagnosis challenging without imaging. Typical manifestations include progressive dyspnea, fatigue, peripheral edema, and signs of heart failure, especially with preserved ejection fraction [23,24]. Additional findings such as orthostatic hypotension, syncope, and arrhythmias are also common [25]. On physical examination, elevated jugular venous pressure, hepatomegaly, and peripheral edema may be observed. Carpal tunnel syndrome, biceps tendon rupture, and lumbar spinal stenosis are clinical red flags that may suggest ATTR amyloidosis [26,27] (Table 1).

**Figure 2:**
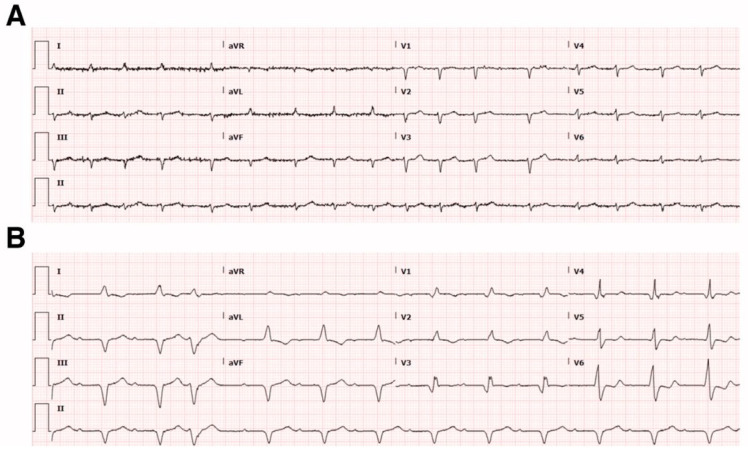
ECGs in a patient with suspected cardiac amyloidosis showing (**A**) low QRS voltages in all leads with atrial fibrillation and (**B**) atrial fibrillation with pseudo-infarct pattern with LBBB.

Electrocardiographic findings include low QRS voltage (limb leads < 5 mm, precordial leads < 10 mm) and a pseudo-infarct pattern, characterized by Q waves in the absence of coronary artery disease [28,29]. These findings, when paired with imaging evidence of left ventricular hypertrophy, raise suspicion for an infiltrative process [30] (Figure 2).

Routine laboratory tests may reveal elevated cardiac biomarkers such as NT-pro BNP and troponins, which are disproportionately elevated compared to the degree of left ventricular systolic dysfunction [31,32]. Serum and urine protein electrophoresis with immunofixation and serum free light-chain assay are essential to evaluate for AL amyloidosis [33].

Given the nonspecific presentation and serious prognostic implications, imaging plays a pivotal role in raising clinical suspicion, prompting further workup, and guiding diagnosis [34,35].

Imaging Testing

- Echocardiogram.
- Most readily available.
- Cheapest option.
- Essentially no contraindications.
- Almost always the first imaging modality to be done, which helps (1) confirm the clinical suspicion of cardiac dysfunction; (2) raise the possibility of diagnosis of cardiac amyloidosis, prompting further workup; (3) provide longitudinal data on cardiac function; and (4) identify complications.
- Cardiac MRI.
- Nuclear imaging.

A study in 2019 by Gilstrap et al. demonstrated the increasing incidence and prevalence of cardiac amyloidosis in men, the elderly, and Black patients, suggesting improved awareness and diagnostic testing through extensive noninvasive imaging [36]. Cardiac amyloidosis should therefore be considered during the initial evaluation of patients > 65 years old hospitalized with heart failure [36].

A multicenter study in 2021 by AbouEzzeddine et al. highlighted the increased prevalence of Transthyretin Amyloid Cardiomyopathy ATTR-CM in a community cohort, with a 2.5% prevalence rate in males and 0% in females [37]. Prevalence increased with age, from 0% in patients aged 60–69 years to 21% in those aged ≥ 90 years (*p* < 0.001). After adjusting for age, ATTR-CM prevalence differed by sex, with a 10.1% rate in men versus 2.2% in women. ATTR-CM was found in a substantial proportion of HFpEF patients with ventricular wall thickening, particularly in older men. These results suggest that systematic evaluation may substantially increase ATTR-CM diagnosis, enabling therapeutically relevant phenotyping of HFpEF [37].

In a study evaluating outcomes of 1230 patients with cardiomyopathy, during a mean follow-up of 4.4 years, Felker et al. (2000) showed that survival was significantly worse among patients with cardiomyopathy due to infiltrative myocardial disease, including cardiac amyloidosis (adjusted hazard ratio, 4.40; 95% confidence interval, 3.04–6.39) [38].

## 4. Echocardiography in Cardiac Amyloidosis

Echocardiography is typically the first-line imaging modality in suspected cardiac amyloidosis due to its accessibility, cost-effectiveness, and ability to provide critical functional and structural information. Consensus guidelines from major societies [American Society of Nuclear Cardiology(ASNC), American Heart Association (AHA), American Society of Echocardiography (ASE), Heart Failure Society of America (HFSA), Society for Cardiovascular Magnetic Resonance (SCMR)] support the role of echocardiography as a foundational tool in the diagnostic algorithm for cardiac amyloidosis [39].

**Figure 3:**
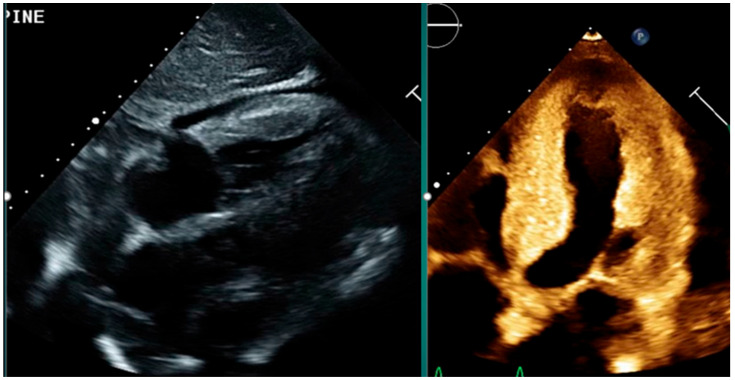
2D ECHO image depicting increased concentric LV wall thickness, granular myocardial texture, thickened valves and small pericardial effusion.

Key echocardiographic findings include:

- Increased left ventricular (LV) wall thickness, often concentric and symmetric.
- Sparkling or granular myocardial texture due to amyloid infiltration.
- Biatrial enlargement and dysfunction.
- Thickened valves and interatrial septum.
- Small pericardial effusions [40] (Figure 3).

**Figure 4:**
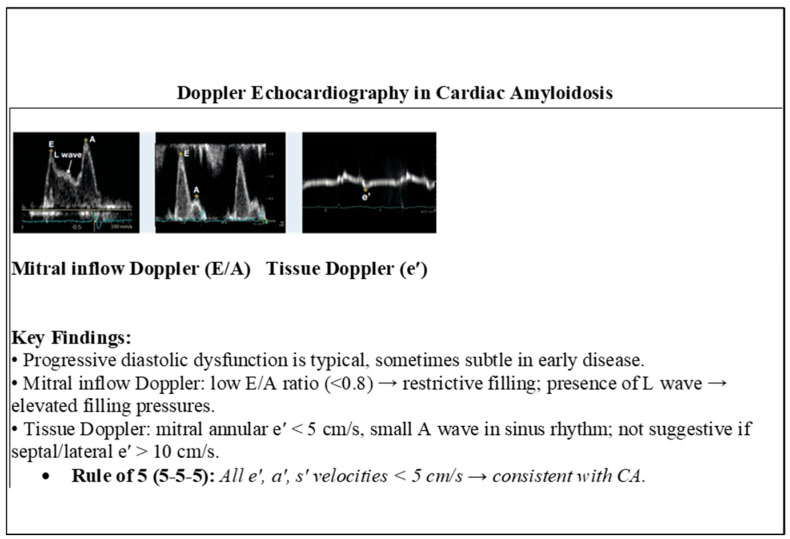
Doppler echocardiography key findings in cardiac amyloidosis.

**Figure 5:**
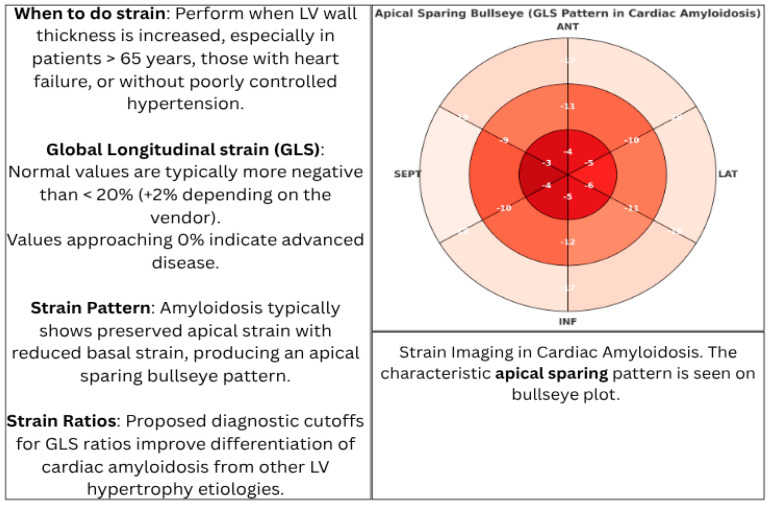
Schematic diagram highlighting the strain imaging pattern (apical sparing) on a bullseye plot in cardiac amyloidosis. Red = severely depressed, Orange = mildly depressed, pink= normal.

Tissue Doppler Imaging (TDI) and strain imaging are especially valuable in early disease detection. A hallmark feature is “apical sparing” of longitudinal strain, also described as the “cherry-on-top” pattern. This finding has demonstrated high sensitivity (93%) and specificity (82%) in identifying cardiac amyloidosis and is an accurate, reproducible method of differentiating cardiac amyloidosis from other causes of LV hypertrophy [41] (Figure 4 and Figure 5).

**Figure 6:**
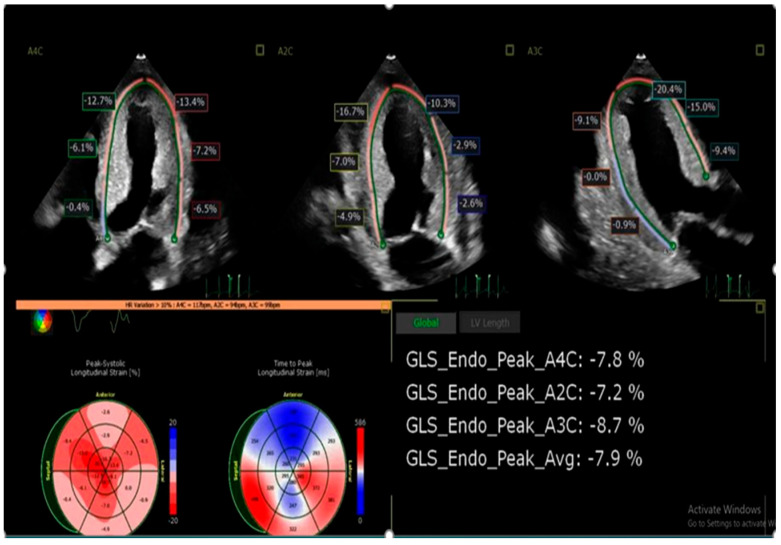
Calculation of LV strain and correlation with bullseye image in patients with cardiac amyloidosis showing “cherry on top” appearance.

Diastolic dysfunction is universal, often progressing from grade I to restrictive filling patterns [42]. Left atrial dysfunction, independent of cavity size, has also been identified as a predictor for atrial arrhythmias [43,44]. Advanced techniques such as myocardial strain analysis and three-dimensional echocardiography are increasingly utilized for risk stratification and monitoring treatment response [45] (Figure 6).

The relationship between ejection fraction (EF) and global longitudinal strain (GLS) in amyloidosis was evaluated in a 2016 study by Pagourelias et al. They proposed the Ejection fraction strain ratio (EFSR = EF/|GLS|) as a novel parameter [46]. This ratio was significantly higher in CA patients compared to controls, with an EFSR cutoff of 4.1 showing strong differentiating capacity for amyloidosis within LV hypertrophy pathologies [46].

Another study assessed the relative regional strain ratio (RRSR), defined as apical LS divided by mid + basal LS. Patients with low EF and high RRSR (>1.19) had the worst prognosis. This tool is both diagnostic and prognostic, with implications for treatment planning [47].

Among patients with AL, standard echocardiographic measures often remain unchanged at 1 year following chemotherapy, despite reductions in cardiac biomarkers. However, longitudinal strain (LS) is a sensitive marker of pre-treatment cardiac dysfunction, predicts survival beyond biomarkers, and detects early improvement following chemotherapy [48].

Left atrial (LA) structure and function are also impaired in CA. A study evaluating LA strain and strain rate demonstrated significant reductions across all phases (reservoir, conduit, active function) compared to matched controls, independent of LA size, EF, and LV filling pressures [49].

Similarly, in hereditary ATTR amyloidosis, LA function is abnormal irrespective of cavity size, and reduced LA myocardial strain rate during atrial systole is a strong predictor of atrial arrhythmias [50].

Finally, the prevalence and prognostic impact of aortic stenosis (AS) in CA have been evaluated. Among patients with CA, ATTRwt was associated with a higher prevalence of AS compared to ATTRv or AL. However, moderate or greater AS was not associated with worsened outcomes in ATTRwt patients [51].

## 5. Cardiac MRI in Cardiac Amyloidosis

Cardiac magnetic resonance imaging (CMR) has emerged as a powerful tool in the evaluation of cardiac amyloidosis due to its superior tissue characterization and ability to detect myocardial infiltration. It is particularly useful in differentiating cardiac amyloidosis from other causes of left ventricular hypertrophy [52].

**Figure 7:**
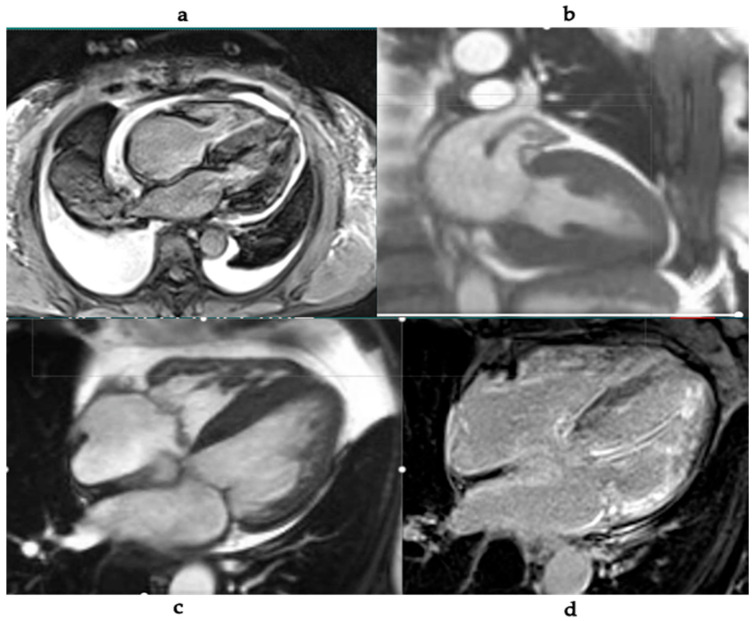
CMR images in cardiac amyloidosis: (**a**) Short-axis cine image showing concentric LV hypertrophy and restricted ventricular cavity due to fibrotic thickening. (**b**) Long-axis cine view illustrating endocardial thickening with preserved basal contractility and apical restriction. (**c**) Four-chamber view showing biatrial enlargement suggestive of diastolic restriction. (**d**) Late gadolinium enhancement (LGE) image revealing dense subendocardial enhancement of the apical and mid-ventricular regions, consistent with cardiac amyloidosis.

Key CMR findings include:

- Increased LV wall thickness and biatrial enlargement.
- Abnormal myocardial nulling pattern on late gadolinium enhancement (LGE).
- Diffuse subendocardial or transmural LGE patterns.
- Elevated native T1 values and increased extracellular volume (ECV) fraction.
- Presence of intracardiac thrombi and small pericardial effusions [53,54] (Figure 7).

LGE imaging is central to diagnosis, with typical findings including global subendocardial or transmural enhancement. Studies have shown that the extent of enhancement correlates with disease severity and prognosis [55]. Native T1 and ECV mapping further aid in quantifying myocardial involvement and tracking response to therapy [56,57]. CMR also offers excellent reproducibility for assessing cardiac morphology and function, enabling longitudinal monitoring. It is considered particularly helpful when echocardiographic findings are inconclusive [58].

In a 2015 study, the authors demonstrated that in a large cohort of AL and ATTR amyloidosis, patients with positive LGE had worse prognosis than those without [55]. Transmural LGE was associated with the greatest amyloid burden and the poorest survival, independent of other risk factors. Phase-sensitive inversion recovery (PSIR) imaging resolved nulling issues and improved diagnostic accuracy. Importantly, three LGE patterns were described: no LGE, subendocardial LGE, and transmural LGE, with prognosis worsening across this spectrum [55].

Parametric mapping with CMR now permits visualization and quantification of changes in myocardial composition based on T1, T2, T2*, and ECV relaxation times [59]. These techniques improve diagnostic precision, inter-patient comparability, and therapeutic monitoring.

CMR with LGE has demonstrated characteristic subendocardial “tramline” enhancement, which may progress to transmural LV and RV involvement in advanced stages [60]. However, atypical LGE distributions are reported, and renal impairment in amyloidosis often limits gadolinium use [60,61,62]. Native T1 mapping offers a promising non-contrast alternative, though reference ranges in chronic kidney disease require further validation [60,61,62].

ECV quantification serves as a surrogate marker of amyloid burden and carries independent prognostic value [63]. Both native T1 and ECV are elevated in early disease, often before LGE becomes apparent, highlighting their value in early detection [63]. ATTR amyloidosis typically demonstrates higher ECV, while AL tends to have higher native T1 values [64].

In a 2017 consensus statement, Messroghli et al. emphasized the value of standardized mapping techniques for inter-center reproducibility [65]. More recently, Martinez-Naharro et al. (2022) showed that changes in ECV track amyloid burden and correlate independently with prognosis after adjusting for other predictors [66]. In a subsequent study, the same group reported that serial native T1 mapping can monitor treatment response without contrast use, with reduced scan time and improved workflow efficiency [67].

Thus, beyond diagnosis, ECV and T1 mapping provide biomarkers for longitudinal assessment of therapy, prognostication, and monitoring of treatment efficacy [66,67].

## 6. Nuclear Imaging in Cardiac Amyloidosis

Radionuclide imaging plays a pivotal role in diagnosing transthyretin (ATTR) amyloidosis, particularly in distinguishing it from AL amyloidosis without the need for biopsy [68]. Consensus guidelines from professional societies, including the American Society of Nuclear Cardiology (ASNC), endorse its central role in the noninvasive diagnostic algorithm for ATTR-CM [69].

**Figure 8:**
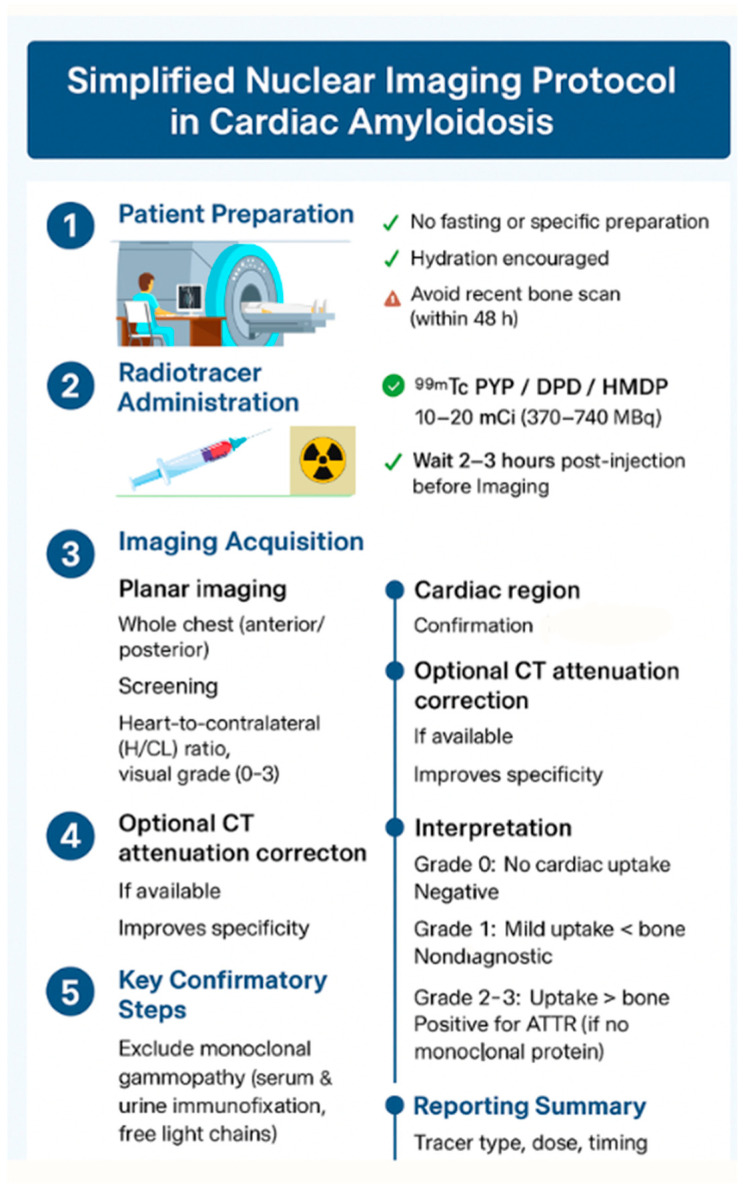
Simplified nuclear imaging protocol in cardiac amyloidosis.

Imaging Modalities include:

- Planar scintigraphy and SPECT using bone-seeking tracers such as ^99m^Tc-pyrophosphate (PYP), 3,3-diphosphono-1,2-propanodicarboxylic acid (DPD), or hydroxymethylene diphosphonate (HMDP).
- SPECT/CT, which provides improved anatomical localization and helps distinguish blood pool activity from true myocardial uptake.
- PET imaging with amyloid-specific tracers (e.g., ^18^F-florbetapir, ^18^F-florbetaben, ^11^C-PiB), which allow high-resolution molecular imaging of amyloid deposits [70,71] (Figure 8).

Planar imaging is simple and widely available. Myocardial uptake is quantified by calculating the heart-to-contralateral chest (H/CL) ratio, with values > 1.5 suggestive of ATTR amyloidosis [72]. Visual grading against rib uptake is also used. Whole-body planar imaging may provide adjunctive evidence of systemic amyloid burden, such as tracer uptake in the shoulder or hip girdles, a specific feature of systemic ATTR amyloidosis [73].

**Figure 9:**
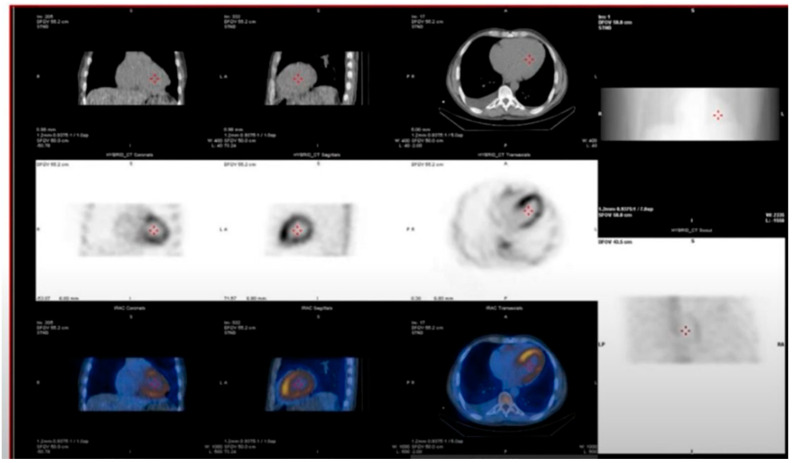
SPECT-CT imaging in cardiac amyloidosis. The middle row and bottom row images demonstrate intense myocardial tracer uptake on SPECT corresponding to the left ventricular myocardium on CT (upper row), with activity equal to or greater than bone uptake. This finding is consistent with a positive ^99m^Tc-pyrophosphate (PYP) scan, characteristic of transthyretin (ATTR) cardiac amyloidosis.

SPECT and SPECT/CT add three-dimensional detail, enabling separation of blood pool activity from myocardial signal and better visualization of uptake in the interventricular septum, which is commonly involved in amyloidosis [74] (Figure 9).

Bone scintigraphy with tracers such as PYP, DPD, and HMDP has shown high specificity and positive predictive value for ATTR amyloidosis when combined with negative monoclonal protein studies [75]. This approach now enables non-biopsy diagnosis of ATTR-CM in the majority of patients [76].

PET amyloid imaging, although less widely available, offers superior spatial resolution and quantification of amyloid burden. ^18F-florbetapir has demonstrated utility in detecting cardiac amyloid deposits even at early stages, particularly in AL amyloidosis [77]. ^11^C-PiB PET has also successfully visualized cardiac amyloid, showing greater uptake in AL compared to ATTR amyloidosis [78].

The adoption of bone-avid tracer scintigraphy for ATTR-CM has grown substantially over the past decade. Successive ASNC surveys have shown increased utilization, broader adoption by clinicians across specialties, and evolving imaging protocols [79].

Thus, radionuclide imaging is now firmly established as a cornerstone modality in the diagnosis and risk stratification of cardiac amyloidosis, with bone scintigraphy enabling noninvasive diagnosis of ATTR-CM and PET imaging providing complementary insights into amyloid biology.

## 7. Current Guidelines and Consensus Recommendations

The increasing availability and accuracy of multimodality imaging techniques have been incorporated into contemporary guidelines for the diagnosis of cardiac amyloidosis. Several professional societies have issued expert consensus statements to standardize the diagnostic approach [80].

**Figure 10:**
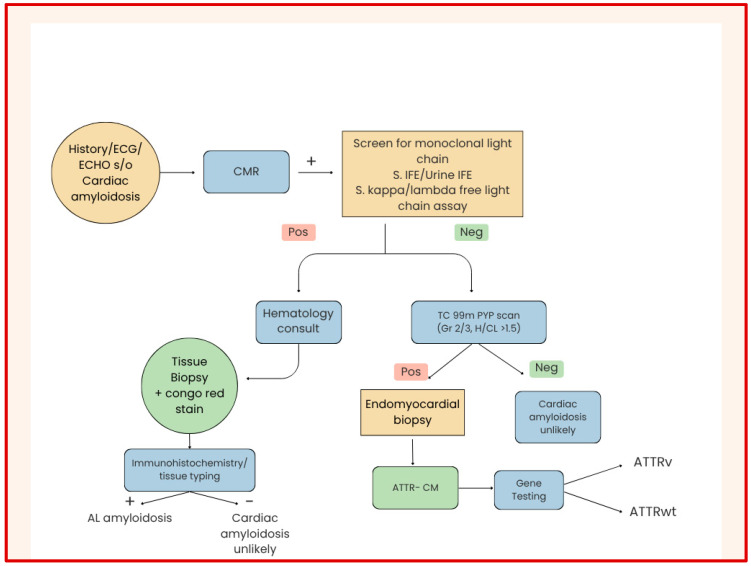
Algorithmic approach to a suspected case of cardiac amyloidosis. + = Positive/present; − = Negative/absent.

The 2021 international expert consensus document by ASNC, AHA, ASE, EANM, HFSA, ISA, SCMR, and SNMMI recommends the following [81]:

- Initial Screening: Begin with clinical suspicion based on symptoms and red flags; perform serum/urine protein electrophoresis and free light-chain assay.
- Echocardiography: As the first-line imaging modality, assess for structural and functional abnormalities, including apical sparing on strain imaging.
- CMR: Recommended when echocardiographic findings are inconclusive or when more detailed tissue characterization is needed.
- Radionuclide Imaging: ^99m^Tc PYP, DPD, or HMDP scintigraphy is recommended for patients without evidence of monoclonal gammopathy to confirm ATTR amyloidosis (Figure 10 and Table 2).

If bone scintigraphy demonstrates Grade 2 or 3 myocardial uptake with no evidence of plasma cell dyscrasia, a non-biopsy diagnosis of ATTR amyloidosis can be established [82].

These guidelines promote a non-invasive, algorithmic approach, reducing the need for endomyocardial biopsy in most patients and improving diagnostic confidence.

The integration of multimodality imaging findings with laboratory and clinical data ensures accurate subtype differentiation and guides appropriate therapy [80,81,82] (Table 3).

## 8. Practical Considerations, Pitfalls, and Recent Advances

Despite the high diagnostic accuracy of bone-avid radiotracers such as ^99mTc-PYP, DPD, and HMDP for transthyretin cardiac amyloidosis (ATTR-CM), several pitfalls may lead to false-positive or false-negative results. False-positive uptake may occur in conditions associated with myocardial injury, including acute myocardial infarction, myocarditis, pericarditis, or significant left ventricular hypertrophy unrelated to amyloidosis [83]. Blood pool activity—particularly in patients with atrial fibrillation, renal dysfunction, or low cardiac output—can mimic myocardial uptake on planar imaging, underscoring the importance of SPECT confirmation to ensure true myocardial localization [84]. Rib fractures, valve calcification, and overlapping skeletal uptake may also contribute to false-positive results [85]. Importantly, the presence of a monoclonal protein can confound interpretation, as AL amyloidosis occasionally demonstrates low-grade tracer uptake; in such cases, radionuclide scintigraphy cannot reliably differentiate AL from ATTR, and biopsy is required [83].

Conversely, false-negative results may occur in early or minimal amyloid infiltration, particularly in certain hereditary ATTR variants with low tracer affinity [86]. Patients with AL amyloidosis may also show absent or minimal uptake because bone tracers exhibit much weaker binding to AL fibrils [83]. Technical limitations—including inadequate imaging delay (e.g., imaging before sufficient blood pool clearance), improper region-of-interest selection, or attenuation artifacts—can further reduce sensitivity [84,87]. In rare cases of advanced diffuse infiltration, near-equal myocardial and skeletal uptake may diminish contrast on planar imaging, leading to under-recognition of disease [88].

Given these potential pitfalls, bone scintigraphy results must always be interpreted alongside monoclonal protein testing, echocardiographic or CMR findings, and the overall clinical context to avoid misclassification and ensure accurate amyloid subtype differentiation.

Although advances in multimodality imaging have dramatically reduced the need for tissue biopsy in many patients with suspected cardiac amyloidosis, biopsy remains essential in selected cases. When a patient has strong clinical, laboratory, or imaging features suggestive of amyloidosis, yet radionuclide scintigraphy, echocardiography, or CMR findings are inconclusive or negative, an endomyocardial biopsy should be pursued to avoid missed or delayed diagnosis [89]. This scenario is particularly relevant in AL amyloidosis, where bone scintigraphy may be negative or equivocal due to the lower affinity of bone-avid tracers for AL fibrils [76,83]. Additionally, certain ATTRv mutations and early-stage ATTR disease may exhibit minimal or absent uptake on bone scintigraphy, leading to false-negative noninvasive results [86].

Biopsy is also indicated when a monoclonal gammopathy is present, since noninvasive imaging cannot reliably exclude AL cardiac amyloidosis in this setting. Misclassification of AL as ATTR has major therapeutic consequences, given the urgency of chemotherapy-based treatment for AL amyloidosis. In such cases, either endomyocardial biopsy or biopsy of an involved extracardiac organ (e.g., fat pad, kidney, or bone marrow with mass spectrometry) is critical for definitive amyloid typing [90].

Ultimately, when the pre-test probability of cardiac amyloidosis remains high—based on clinical red flags (e.g., intolerance to heart failure therapy, neuropathy, bilateral carpal tunnel syndrome), biomarker profile, or imaging patterns—histologic confirmation becomes a key step to ensure accurate subtype classification and guide appropriate therapy.

A significant recent advancement in cardiac amyloidosis imaging is the development of 124I-evuzamitide, a pan-amyloid–binding PET tracer designed to detect amyloid deposits across multiple organs. In a phase 1/2 study, 124I-evuzamitide PET/CT demonstrated high sensitivity (≈93.6%) for detecting cardiac amyloid, with uptake observed in both ATTR and AL amyloidosis. Importantly, myocardial tracer uptake correlated with clinical manifestations and cardiac involvement, supporting its potential diagnostic and prognostic utility [91]. In several cases, evuzamitide identified cardiac amyloid deposition even when conventional bone-avid scintigraphy (e.g., ^99mTc-PYP) was negative, suggesting greater sensitivity for early or subtle myocardial infiltration [92].

Based on these promising findings, the U.S. Food and Drug Administration granted Breakthrough Therapy Designation for evuzamitide as a diagnostic imaging agent for suspected cardiac amyloidosis, with additional Orphan Drug Designations from both the FDA and the European Medicines Agency [93]. As a pan-amyloid tracer, evuzamitide enables whole-body visualization of amyloid burden, including cardiac, renal, hepatic, splenic, and soft-tissue involvement, providing a comprehensive assessment of systemic disease [94]. If validated in larger multicenter studies, 124I-evuzamitide may significantly enhance early detection, facilitate disease monitoring, and refine therapeutic decision-making by quantifying global amyloid load.

In a comprehensive State-of-the-Art Review, Rapezzi et al. critically compared contemporary guidance documents from major international societies—including ASNC, AHA, ESC, HFSA, ISA, and SCMR—and highlighted both areas of agreement and important discrepancies within current recommendations for the diagnosis and management of cardiac amyloidosis [95]. The review notes strong consensus across societies regarding the need for heightened clinical awareness, early recognition of red-flag features, and the central role of multimodality imaging—particularly echocardiography, CMR, and bone-avid nuclear scintigraphy—in diagnostic pathways. All societies universally emphasize differentiating AL from ATTR amyloidosis, reflecting fundamental differences in treatment urgency, therapeutic strategies, and prognosis. However, the authors underscore meaningful variations between guidelines in the sequencing of tests, interpretation of radionuclide scintigraphy (particularly Grade 1 uptake), and thresholds for advancing to endomyocardial biopsy, especially when a monoclonal gammopathy is present [96,97]. Terminology for diagnostic certainty (“suspected,” “probable,” “definite” amyloidosis) and criteria defining myocardial involvement also differ subtly among professional societies, potentially contributing to inconsistent diagnostic approaches and variability in clinical decision-making. Additionally, the review highlights uneven integration of emerging modalities—such as T1/ECV mapping, strain imaging, and amyloid-specific PET tracers—across society documents, despite growing evidence supporting their diagnostic and prognostic value [98]. Rapezzi and colleagues argue that these discrepancies may delay diagnosis, increase misclassification risk, and complicate implementation of evolving therapeutic strategies, particularly in regions with differing imaging availability. Accordingly, the authors call for greater international harmonization, proposing that future guidelines adopt unified terminology, standardized imaging criteria, and algorithmic pathways that fully integrate both established and emerging technologies to improve diagnostic precision and patient outcomes [95,96,97,98]. Important key studies in cardiac amyloidosis imaging have been summarized in the reference table below [Table 4].

## 9. Conclusions

Cardiac amyloidosis, once considered rare, is now increasingly diagnosed due to advances in multimodality imaging and heightened clinical awareness. Differentiating between AL and ATTR subtypes is essential for appropriate therapy, and this is best achieved through an integrated diagnostic strategy.

Echocardiography remains the frontline modality, while cardiac MRI adds vital tissue characterization, and nuclear imaging—especially with bone-avid tracers—has revolutionized non-invasive diagnosis of ATTR. These tools, when used in combination and guided by current consensus recommendations, offer high diagnostic accuracy and enable early therapeutic intervention.

Ongoing advancements in imaging techniques and therapeutic options continue to improve outcomes for patients with cardiac amyloidosis. Multidisciplinary collaboration and adherence to standardized algorithms will be key in optimizing care.

## Figures and Tables

**Table 1:** Summary of clinical features and imaging findings.

Category	Clinical Features/Findings
Clinical Features	- Heart failure in adults aged >60 y - Significant aortic stenosis in adults aged >65 y - Hypotension or normotension if previously hypertensive individual - Peripheral or autonomic neuropathy - Bilateral carpal tunnel syndrome - Lumbar spinal stenosis - Biceps tendon rupture - Family history of cardiomyopathy - Hypertrophic cardiomyopathy in older adults - History of multiple orthopedic surgeries (hip, knee, shoulder)
Electrocardiogram (ECG)/Cardiac Imaging Findings	Increased left ventricular wall thickness above normal for sex and clinical features above with any of the following: - Discordance between QRS voltage on ECG and wall thickness - Low voltage complexes - Pseudo-infarcts on ECG - Atrioventricular conduction disease - Restrictive phenotype: small chambers and enlarged atrium - Increased atrial septal thickness - Reduced longitudinal strain with apical sparing - Low tissue Doppler velocities - Late gadolinium enhancement on cardiac MRI - Increased extracellular volume fraction - Increased native myocardial T1 time

**Table 2:** Diagnostic criteria in cardiac amyloidosis [80,81,82].

Category	Criteria	Amyloidosis Type
Clinical Diagnosis of ATTR Cardiac Amyloidosis	ATTR cardiac amyloidosis is diagnosed when all of the following are met: 99mTc-PYP, DPD, or HMDP Grade 2 or 3 myocardial uptake of radiotracer AND Absence of a clonal plasma cell process as assessed by serum FLCs, and serum and urine immunofixation AND Typical cardiac imaging features (see below)	ATTR
Typical Imaging Features of Cardiac Amyloidosis	Echocardiography: - LV wall thickness > 12 mm - Relative apical sparing of global LS ratio (average of apical LS/average of combined mid + basal LS > 1) - ≥Grade 2 diastolic dysfunction CMR: - LV wall thickness > upper limit of normal for sex on SSFP cine CMR - Global ECV > 0.40 - Diffuse LGE—Abnormal gadolinium kinetics typical for amyloidosis, with myocardial nulling prior to blood pool nulling PET (^18^F-florbetapir or ^18^F-florbetaben PET): - Target-to-background (LV myocardium to blood pool) ratio > 1.5 - Retention index > 0.030 min^−1^	ATTR/AL

**Table 3:** Summary of role of different imaging modalities in cardiac amyloidosis.

Imaging Modality	Key Features	Clinical Utility	Limitations
Echocardiography	- Increased LV wall thickness- Granular myocardial texture- Pericardial effusion- Apical sparing on strain- LA dysfunction on tissue Doppler	- First-line imaging- Widely available- Assesses structure and function- Suggests amyloid pattern	- Non-specific findings- Limited tissue characterization
Strain Imaging (GLS)	- Relative apical sparing (“cherry-on-top”)- Longitudinal strain analysis	- High sensitivity/specificity for amyloidosis- Early myocardial dysfunction assessment	- Strain abnormalities may overlap with other cardiomyopathies
Tissue Doppler Imaging	- Reduced LA strain- Abnormal diastolic parameters	- Helps assess atrial mechanical function- Predictor of arrhythmias	- Operator dependent- May not be routinely performed in all labs
Cardiac MRI (CMR)	- LGE (subendocardial/transmural)- T1/T2 mapping- ECV quantification- Detection of thrombi, effusions	- Excellent tissue characterization- Staging and prognosis- Quantitative assessment of amyloid burden	- Gadolinium contraindicated in advanced CKD- Cost and availability
Bone Scintigraphy (99mTc-PYP/DPD/HMDP)	- Myocardial uptake graded visually and with H/CL ratio- SPECT/CT for localization	- Confirms ATTR in absence of monoclonal protein- High sensitivity and specificity	- Cannot differentiate ATTRwt vs. ATTRv- False positives in AL if protein not ruled out
PET Imaging (e.g., 18F-florbetapir, 11C-PiB)	- Direct visualization of amyloid fibrils- SUV-based quantification	- Early detection- Subtype differentiation (under study)- Whole-body amyloid burden assessment	- Experimental- Limited tracer availability- Expensive

**Table 4:** Reference table of key studies in cardiac amyloidosis imaging.

Study	Imaging Modality	Key Findings	Clinical Implication
Phelan et al. [8]	Echo (Strain)	Relative apical sparing of longitudinal strain showed 93% sensitivity and 82% specificity	Diagnostic hallmark of cardiac amyloidosis
Fontana et al. [9]	CMR	No LGE associated with 92% survival vs. 61% with transmural LGE	LGE pattern predicts prognosis
Felker et al. [39]	Prognosis	Median survival <6 months in untreated AL-CA	Highlights urgency for early diagnosis
Pagourelias et al. [46]	Echo (Strain)	Regional strain ratio differentiates amyloidosis from HCM	Aids in differential diagnosis
Senapati et al. [47]	Echo (Strain)	Strain improvement correlated with survival in AL amyloidosis	Useful for monitoring treatment response
Salinaro et al. [48]	Echo (Strain)	Longitudinal strain improvement post-treatment linked with outcomes	Prognostic indicator in AL amyloidosis
Syed et al. [52]	CMR	Differentiated AL vs. ATTR based on LGE pattern	Helps in amyloid typing
Messroghli et al. [65]	CMR (Mapping)	ECV and T1 mapping accurately quantify amyloid	Quantitative non-invasive staging
Martinez-Naharro et al. [66]	CMR	ECV elevation linked with disease severity	Guides disease staging and therapy
Ruberg et al. [99]	Epidemiology	Increased recognition and prevalence of ATTR-CA	Raises awareness of underdiagnosis
Martinez-Naharro et al. [100]	CMR, PYP	Imaging burden associated with adverse outcomes	Supports integrated imaging risk stratification
Jerome et al. [101]	Nuclear Imaging	Guidelines for PYP imaging, SPECT, and interpretation	Standardized non-invasive diagnostic workflow
Bullock-Palmer et al. [102]	PYP SPECT	99mTc-PYP uptake highly specific for ATTR in absence of monoclonal protein	Enables non-biopsy diagnosis
Hotta et al. [103]	PET	Tracer uptake (^18^F-florbetapir, ^11^C-PiB) visualizes amyloid	Foundation for PET-based evaluation
Ozbay et al. [104]	Echo (Tissue Doppler), PYP SPECT	Primary LA cardiomyopathy associated with thromboembolism independent of AF and CHA_2_DS_2_-VASc	LA strain and stiffness identify ATTR-CM independent of AF and diastolic dysfunction

## Data Availability

No new data were created or analyzed in this study. Data sharing is not applicable to this article.

## Abbreviations

Abbreviation	Definition
AL	Light-chain amyloidosis
ATTRwt	Wild-type transthyretin amyloidosis
ATTRv	Variant (hereditary) transthyretin amyloidosis
ATTR-CM	Transthyretin Amyloid Cardiomyopathy
CMR	Cardiac magnetic resonance
LGE	Late gadolinium enhancement
ECV	Extracellular volume fraction
SPECT	Single-photon emission computed tomography
PET	Positron emission tomography
PYP	Pyrophosphate
NT-proBNP	N-terminal pro–B-type natriuretic peptide
LVEF	Left ventricular ejection fraction
T1/T2	Relaxation times on CMR
SUV	Standardized uptake value
GLS	Global longitudinal strain
HFpEF	Heart failure with preserved ejection fraction
